# Development and Validation of a UHPLC–MS/MS Method for Quantitation of Almonertinib in Rat Plasma: Application to an *in vivo* Interaction Study Between Paxlovid and Almonertinib

**DOI:** 10.3389/fphar.2022.960311

**Published:** 2022-07-22

**Authors:** Peng-fei Tang, Su-su Bao, Nan-yong Gao, Chuan-feng Shao, Wei-fei Xie, Xue-meng Wu, Le-ping Zhao, Zhong-xiang Xiao

**Affiliations:** ^1^ Affiliated Yueqing Hospital, Wenzhou Medical University, Wenzhou, China; ^2^ Department of Pharmacology, School of Pharmacy of Wenzhou Medical University, Wenzhou, China; ^3^ Market Supervision Administration of Yueqing City, Wenzhou, China

**Keywords:** almonertinib, drug-drug interaction, UHPLC-MS/MS, Paxlovid, pharmacokinetics

## Abstract

Almonertinib was approved for the first-line treatment of advanced NSCLC patients with EGFR-TKI-sensitive genetic mutations by National Medical Products Administration (NMPA) in 2021.The purpose of this study was to establish and validate a fast, accurate, stable and facile ultra-performance liquid chromatography-tandem mass spectrometry method for the quantification of almonertinib in rat plasma, it was employed to explore the effect of Paxlovid on the pharmacokinetics of almonertinib in rats. Zanubrutinib was used as an internal standard (IS), and the plasma samples were prepared by the protein precipitation method using acetonitrile. Chromatographic separation was carried out on a Shimadzu LC-20AT ultra-performance liquid chromatography system using a Shim-pack velox C18 (2.1× 50 mm, 2.7 μM) column. The mobile phase consisted of methanol and 0.1% formic acid-water. Mass spectrum analysis was executed using Shimadzu 8040 Triple quadrupole mass spectrometry. The precursor and product ions of the analyte and internal standard were detected in multiple reaction monitoring (MRM) mode. The typical fragment ions were *m/z 526.20 → 72.10* for almonertinib and *m/z 472.15 → 290.00* for zanubrutinib (IS). The method was validated to have good linearity for quantifying almonertinib in rat plasma from 0.1–1000 ng/ml (R^2^ = 0.999), and the LLOQ was 0.1 ng/ml. The validity of this method was sufficiently verified for selectivity, specificity, extraction recovery, matrix effect, accuracy, precision and stability. The validated UHPLC–MS/MS method was successfully applied to the drug interaction study of almonertinib with Paxlovid in rats. Paxlovid significantly inhibits the metabolism of almonertinib and increased the exposure of almonertinib. This study can help us to understand the metabolic profile of almonertinib better, and further human trials should be conducted to validate the results.

## 1 Introduction

**FIGURE 1:**
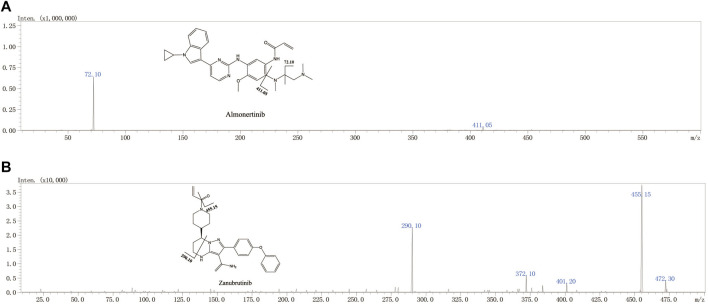
The chemical structures and Mass spectra of almonertinib and IS in the present study.

Almonertinib is a new type of irreversible third-generation EGFR inhibitor, that is, highly selective for EGFR-TKI-sensitive genetic mutations and T790 M drug-resistant gene mutations (Nagasaka et al., 2021). Almonertinib was approved for the treatment of patients with advanced NSCLC following progression on prior EGFR-TKIs and having a T790M drug-resistance mutation by the National Medical Products Administration (NMPA) in 2020 (Lu et al., 2021). It was subsequently approved for the first-line treatment of advanced NSCLC patients with EGFR-TKI-sensitive genetic mutations by NMPA in 2021 based on the AENEAS trial (Lu, 2021). In contrast to earlier generations of EGFR-TKIs, almonertinib is a small molecular drug that uses pyrimidine as a structural basis and retains the acrylamide structure; it exerts antitumor effects by covalently binding cysteine 797 at the ATP binding site of the TK domain (Yang et al., 2020). (Figure 1) Compared with the first-generation EGFR-TIK gefitinib, almonertinib has shown obvious advantages in progression-free survival (PFS), duration of response (DOR) and objective response rate (ORR) for first-line therapy (Lu, 2021). The higher selectivity for EGFR-TKI-sensitive genetic mutations and relatively lower off-target effects for other wild-type EGFRs mean that almonertinib shows better security than previous generations (Lu, 2021; Shirley and Keam, 2022). The AENEAS results show that the incidence of drug-related adverse reactions was lower with almonertinib than with gefitinib for mild adverse events, such as rash (23.4% vs. 41.4%), diarrhea (16.4% vs. 35.8%), liver damage (29.9% vs. 54.0%) and serious adverse events (4.2% vs. 11.2%) (Lu, 2021).

Beginning in 2019, COVID-19 caused by the SARS-CoV-2 pose a constant threat to global people’s health (Pollard et al., 2020). Paxlovid was approved for the treatment of adults and pediatric patients with mild-to-moderate COVID-19 by the FDA emergency use authorization in December 2021 (Saravolatz et al., 2022). Paxlovid consist of nirmatrelvir, an antiviral active compound against SARS-CoV-2 and ritonavir, a CYP3A4 inhibitor (Owen et al., 2021). For the patient with both NSCLC and COVID-19, drug therapy requires the combination of almonertinib and Paxlovid (Passaro et al., 2021). The results of *in vitro* experiments show that almonertinib is primarily metabolized to N-demethylation metabolite through CYP3A4 (Liu et al., 2022a). In clinical study, the combination of almonertinib and CYP3A4 inducers or inhibitors could have affected plasma concentration of almonertinib and thus influences clinical effect and adverse reactions of almonertinib (Liu et al., 2022a). Therefore, it is necessary to establish a technique for the fast and accurate determination of almonertinib concentrations in plasma, which will help to evaluate the change of pharmacokinetics parameters and discover potential drug interactions during combined administration.

To our knowledge, only one analytical method to quantify almonertinib in biological sample have been reported (Liu et al., 2022b). This method is less than perfect for a number of reasons. For example, it is only applicable in human plasma, has a narrow linear range, and lacks sufficient experimental data validated by other laboratories. Thus, this UHPLC-MS/MS method can’t fulfill the requirements of the preclinical drug-drug interaction studies. Therefore, the purpose of this study was to establish and validate a fast, accurate, stable UHPLC-MS/MS method for the quantification of almonertinib in rat plasma. The feasibility and accuracy of this method are verified by selectivity, specificity, extraction recovery, matrix effect, accuracy, precision and stability experiments. Finally, the effects of Paxlovid on drug exposure and pharmacokinetic parameters of almonertinib were assessed using established methods.

## 2 Materials and Methods

### 2.1 Chemicals and Reagents

Almonertinib (over 99% purity), Nirmatrelvir (purity 99.0%), Ritonavir (purity 99.0%) and the internal standard (IS) Zanubrutinib (purity 99.0%) were purchased from Toronto Research Chemicals Inc. (Toronto, Ontario, Canada). Analytical-grade methanol, acetonitrile, water and formic acid for mass spectrometry were obtained from Honeywell Burdick & Jackson (Muskegon, Michigan, United States). Other reagents used were purchased from J&K Scientific Ltd. (Shanghai, China).

### 2.2 UHPLC–MS/MS Detection Method

Sample chromatographic separation was carried out on a Shimadzu LC-20AT ultra-performance liquid chromatography system (Shimadzu Corp., Tokyo, Japan), which was equipped with a vacuum degas unit, an infusion pump, an autosampler and a column oven. A Shim-pack velox C18 (2.1× 50 mm, 2.7 μM) column at 40°C was used to perform the separation process. After a long period of exploration, 0.1% formic acid-water (A) and methanol (B) compose the mobile phase. The whole analysis process adopts gradient elution mode, and the elution procedure was used as follows: the first 0–0.5 min maintained at 10% B, the next 0.5–0.51 min linear increase to 80% B, 0.51–1.5 min maintained at 80% B, 1.5–2.0 min linear decrease to 10%, and the last 2.0–3 min maintained at 10%. The total running time of each injection was 3 min. During the analysis process, the flow rate was maintained at 0.4 ml/min, and the sample injection volume was set to 2 μl. Good separating efficacy of almonertinib and zanubrutinib (IS) was observed under the above test conditions, and their retention times were 2.08 and 2.35 min, respectively.

Sample detection was executed using Shimadzu 8040 Triple quadrupole mass spectrometry (Shimadzu Corp., Tokyo, Japan). The instrument was equipped with electrospray ionization (ESI) and dedicated data acquisition workstations. The various parameters of the instrument were set as follows: the detector voltage was set to 4.5 kV, the heating block temperature was set to 400°C, and the flow of atomizing gas and drying gas was 3 L/min and 5 L/min, respectively. The precursor and product ions of the analyte and internal standard were detected in multiple reaction monitoring (MRM) mode. To ensure the specificity of the detection, we used the fragment with the highest signal strength as the quantitation, and the second strength product ions were used for qualification. In addition to specific parent and product ions, the retention time of the analyte and IS was also used to improve the specificity of detection. Detailed MS parameters information for almonertinib and zanubrutinib are listed in Table 1.

**TABLE 1:** MS parameters of almonertinib and zanubrutinib.

Analytes	Precursor ion (*m/z*)	Product ion 1 (*m/z*)	Collision energy 1 (V)	Product ion 2 (*m/z*)	Collision energy 2 (V)
Almonertinib	526.20	72.10	35	411.05	32
Zanubrutinib	472.15	290.00	40	455.15	36

### 2.3 Calibration Solution and Quality Control Samples Preparation

Stock solutions (1 mg/ml) of almonertinib and IS (zanubrutinib) were prepared by dissolving the respective standards in methanol. The stock solution was diluted with methanol by multiple grades, and various concentrations of working solutions were obtained. Sample preparation for each standard curve point and quality control (QC) were performed by adding 10 µl of the corresponding almonertinib working solution to 90 µl of blank rat plasma. The concentrations of all points of the standard curve were finally determined to be 0.1, 0.5, 1, 5, 10, 50, 100, 500, and 1000 ng/ml. The working solution of the IS (internal standard) was prepared by diluting the stock solution with methanol to a final concentration of 400 ng/ml. Quality control (QC) samples of three concentrations (0.3, 100, and 800 ng/ml) were prepared the same way. All samples and solutions were placed in a medical freezer for cold storage at −20°C and transferred to room temperature before determination.

### 2.4 Sample Preparation

The rat plasma samples were transferred from a −80°C medical refrigerator to room temperature for thawing before preparation. Then, 100 µl plasma and 40 µl internal standard were added to a 1.5 ml centrifuge tube, and 200 µl acetonitrile was added for protein precipitation. The centrifuge tube was placed on the vortex for 2 min to achieve perfect mixing. The mixture was centrifuged at 12,000 g for 10 min, and then 100 µl supernatant was transferred to a new centrifuge tube containing 100 µl ultrapure water. After gently mixing the centrifuge tube for 30 s, the mixture was used for UHPLC–MS/MS analysis.

### 2.5 Method Validation

Before using the present method for detection, the linearity, stability, selectivity, recovery, accuracy, matrix effect and precision of the method were verified according to FDA method validation guidance (U.S. Department of Health and Human Services Food and Drug Administration et al., 2018).

#### 2.5.1 Selectivity and Specificity

Selectivity is the ability of the analytical method to distinguish and accurately quantify the target compound in a mixture. The selectivity of the method was determined by comparing the test results of blank plasma from six different rats, blank plasma containing almonertinib and IS, and rat plasma samples after oral administration.

#### 2.5.2 Linearity, Low Limit of Detection and Lower Limit of Quantification

Standard curves were established by testing standard samples at nine various concentrations (0.1–1000 ng/ml) on three different days. Data from the regression line of the peak area ratios against concentrations can provide mathematical estimates of the degree of linearity. The low limit of detection (LLOD) is the lowest concentration of an analyte that an analytical method can reliably measure above that of a blank at the 99% confidence level. LLOD was evaluated based on the signal-to-noise ratio of at least 3:1. The lower limit of quantification (LLOQ) is the lowest amount of almonertinib that can be quantitatively determined with acceptable precision and accuracy. The LLOQ was evaluated based on the signal to noise ratio of at least 10:1.

#### 2.5.3 Extraction Recovery and Matrix Effect

Extraction recovery and matrix effects were evaluated using blank plasma from six different rats and three different concentrations (0.3, 100 and 800 ng/ml) of almonertinib QC standards. The comparison of the peak areas of QC samples pre-spiked in blank plasma with those of post-extracted blank plasma spiked samples was defined to evaluate the extraction recovery of almonertinib from rat plasma at the same concentrations. Matrix effects were evaluated by comparing the slope of the standard addition plot with the slope of standard calibration plot.

#### 2.5.4 Accuracy and Precision

Rat plasma QC samples at three different concentration levels (0.3, 100 and 800 ng/ml) were measured by the present method on a single day or three different days. The relative error (RE%) and relative standard deviation (RSD%) of the test results should be calculated to determine whether they are within the specified range (15%) and used to evaluate the precision of the present method. The apparent recoveries should be calculated by dividing the measured almonertinib concentrations to the nominal spiked values in the blank matrix to assess accuracy, and the values of apparent recoveries should be between 85% and 115%.

#### 2.5.5 Stability

Rat plasma QC samples at three different concentration levels (0.3, 100,and 800 ng/ml) under various storage conditions were determined in six replicates to study the stability of the method. These studies reflect the stability of QC samples during storage and analysis, including stability in the analysis experiment (4 h at room temperature), short-term storage (4°C for 24 h), long-term storage (−80°C for a month) and freezing-thawing cycles (three times). The RSD% and RE% of the testing results were calculated, and values below 15% and ±15% were thought to be stable.

### 2.6 DDI Study

Twelve male Sprague–Dawley (SD) rats (200 ± 20 g) were supplied by the Animal Experiment Center of Wenzhou Medical University. All animal-related experimental procedures complied with the Guide for Care and Use of Laboratory Animals and were approved by the Animal Research Ethics Committee of Wenzhou Medical University (Ethics approval number: wydw 2021-0019). Before the start of the experiment, all SD rats were raised on a SPF level lab, and received sufficient food and water. Almonertinib, nirmatrelvir and ritonavir were dissolved in 0.5% carboxymethyl cellulose sodium (CMC-Na) solution. The rats were fasted for 12 hours before the pharmacokinetics experiment but not banned from drinking water. All SD rats were split at random into two groups of 6. Group B (experimental group) were given 55 mg/kg nirmatrelvir and 20 mg/kg ritonavir by gavage administration, and Group A (control group) were given the same dose 0.5% CMC-Na. After half an hour, we infused 10 mg/kg almonertinib into the stomach of each rat and then obtained 0.3mlblood from the caudal vein of each rat at different time points (0.25 h, 1 h, 2 h, 3 h, 4 h, 5 h, 6 h, 7 h, 8 h, 10 h, 12 h and 24 h). Blood samples were collected in heparin tubes, and centrifuged at 13,000 g for 10 min at 4°C. Then, plasma was transferred to sterile tubes and stored in a −80°C refrigerator until the testing process.

The pharmacokinetic parameters of almonertinib were calculated by DAS 3.0 software using the noncompartment model. The critical pharmacokinetic parameters of the two groups were conducted in One-way ANOVA and Dunnett’s test by using software SPSS version 17.0. A *p* value less than 0.05 indicates a significant difference between the two groups.

## 3 Results and Discussion

### 3.1 Method Development and Optimization

#### 3.1.1 Chromatographic Condition Development Optimization

**FIGURE 2:**
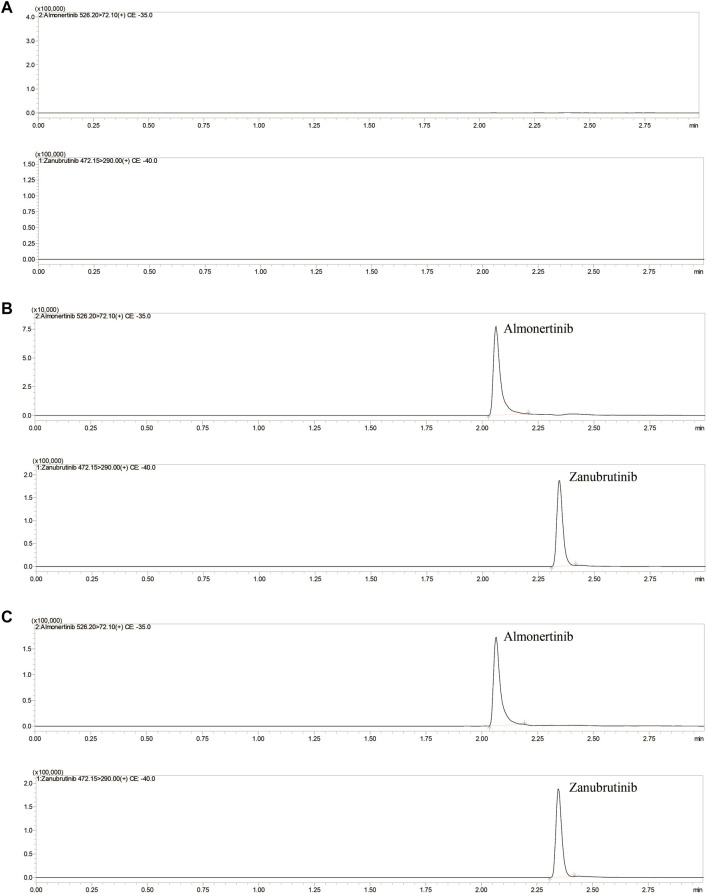
Representative UHPLC–MS/MS chromatograms of almonertinib and zanubrutinib (IS). **(A)** blank plasma; **(B)** a blank plasma sample spiked with almonertinib and IS; **(C)** a rat plasma sample obtained 1 h after oral administration of almonertinib.

The mobile phase composition, elution mode, types of separation columns and temperature of the column were optimized to achieve high-efficiency separation of almonertinib and IS, which facilitates the present method carrying higher sensitivity, specificity, shorter running time and more perfect peak shapes. Performance tests were carried out for different types of columns, such as different column lengths, different particle sizes and different packing materials. A Shim-pack velox C18 (2.1× 50 mm, 2.7 μM) column showed a better chromatographic peak form, retained value and separation. A comprehensive assessment of different mobile phase compositions, such as acetonitrile, methanol, water containing or without 0.1% formic acid or other inorganic salts, was performed. The mobile phase composed of methanol and water containing 0.1% formic acid implemented high separation and a better peak shape. Isocratic or gradient elution, flow rate 0.3–0.5 ml/min and column temperature 20–40°C were tested. Through comparative analyses, suitable types of programs and parameters for the method are selected. Finally, methanol and 0.1% formic acid-water were selected as the mobile phase, and gradient elution was performed under a flow rate of 0.4 ml/min and column temperature of 40°C. The mobile phase ratio was methanol and 0.1% formic acid-water (10:90) at the beginning of the gradient elution program, and then the methanol volume percentage rises to 80% at 0.5 min. Keep the methanol volume percentage at 80% until 1.5 min, and then the percentage of methanol dropped to 10% within half a minute, and finally maintain this ratio until the program finished at 3 min. The total run time of method was 3 min, and retention times of almonertinib and IS were 2.08 and 2.35 min, respectively. Figure 2 shows characteristic chromatograms of blank control, blank rat plasma containing almonertinib and IS standard, and rat plasma after intragastric administration.

#### 3.1.2 Mass Spectrometer Condition Optimization

The running parameters of the mass spectrometer, such as CE (collision energy), detection modes, atomizing gas and drying gas flow rate, and CID (collision-induced dissociation) gas pressure, were optimized to obtain optimal performance for the detection of almonertinib and IS. The optimized parameters should look like this: atomizing gas and drying gas flow rate 3 and 15 L/min, CID gas pressure 17 kPa, DL temperature 250°C. In positive ion mode, CE values are shown in Table 1.

#### 3.1.3 Optimization of Sample Preparation and Internal Standard

The most common methods used for the extraction of compounds in biological samples were the protein precipitation method and solvent extraction. By analyzing and comparing the above two technologies, we found the most suitable method to extract almonertinib and IS. The results reveal that among various kinds of compound extraction methods, acetonitrile-based protein precipitation showed a higher extraction recovery (95.5%–98.0%) and was a simpler and more convenient method. Compared with other precipitants, such as methanol, perchloric acid, ethanol and dimethyl sulfoxide, acetonitrile exhibited better protein precipitation results and more stable chemical properties. For the reasons presented above, we decided to use the acetonitrile precipitation method for sample pretreatment.

To explore the more appropriate IS, classic internal standards (dextromethorphan and nifedipine) and analog internal standards (zanubrutinib and gefitinib) were tested. The results showed that zanubrutinib not only had remarkable stability and sensitivity but also showed similar chemistry and retention time to almonertinib. More importantly, they are all appropriate for the positive ion monitoring mode.

### 3.2 Method Validation

#### 3.2.1 Selectivity and Specificity

Figure 2 shows the chromatograms of blank plasma, blank plasma containing standard preparations of almonertinib and IS, and rat plasma samples after oral administration. The relative retention times were approximately 2.08 min for almonertinib and 2.35 min for IS. The detection method was not interfered with by endogenous substances and commonly used chemicals.

#### 3.2.2 Linearity and Lower Limit of Quantification

Linear regression analysis was performed on the relative peak area (almonertinib/IS) and corresponding serum concentration by the least squares method. L The regression parameters were calculated for the calibration curves. The STEYX of almonertinib was 0.0072. The S_a_ and S_b_ values of almonertinib were 1.4×10^−6^ and 3.2×10^−6^, respectively. STEYX is the standard error of estimation, S_a_ is the standard deviation of the intercept, S_b_ is the standard deviation of the slope. The LLOQ of the almonertinibwas 0.1 ng/ml, and the corresponding RSD and RE were <9.92% and within 0.74%, respectively. The LLOD of our detection method was 0.03 ng/ml. The linearity of the calibration curves was validated by acceptable values of STEYX, S_b_, S_a_, LLOD and LLOQ.

#### 3.2.3 Extraction Recovery and Matrix Effect

The extraction recovery and matrix effects (MEs) of almonertinib QC samples at high, medium and low concentrations (0.3, 100 and 800 ng/ml) are shown in Table 2. The average extraction recoveries of almonertinib at concentrations of 0.3, 100 and 800 ng/ml were 95.7%, 94.1%, and 97.2%, respectively, and the MEs were 98.8%, 99.3%, and 99.0%, respectively. The results of the QC sample test have shown that the detection method has high recovery and that the matrix effects can be ignored in daily determination.

**TABLE 2:** Extraction recovery and matrix effect of almonertinib in rat plasma (*n* = 6).

Analyte	Concentration (ng/ml)	Extraction recovery (%)		Matrix effect (%)	
		Mean ± SD	RSD (%)	Mean ± SD	RSD (%)
Almonertinib	0.3	95.7 ± 5.4	5.6	98.8 ± 8.3	8.4
	100	94.1 ± 5.1	5.4	99.3 ± 6.4	6.5
	800	97.2 ± 4.3	4.5	99.0 ± 10.4	10.5

#### 3.2.4 Accuracy and Precision

Accuracy and precision evaluation of the method was carried out by calculating the apparent recoveries, RE% and RSD% for three concentration levels of QC samples and LLOQ. The end experimental results are displayed in Table 3. The apparent recoveries were between 96.0% and 98.7% at different concentrations. The intra- and interday RSD% values were lower than 10.7% and 6.4%, respectively, and the corresponding RE% values were in the ranges of −5.9% −3.5% and −0.8% −3.0%, respectively. Excellent accuracy and reproducibility of the method have been revealed by experimental data.

**TABLE 3:** Precision and accuracy for almonertinib of QC samples in rat plasma (*n* = 6).

Analyte	Concentration (ng/ml)	Intra-day			Inter-day			Apparent recovery (%)
		Mean ± SD	RSD (%)	RE (%)	Mean ± SD	RSD (%)	RE (%)	
Almonertinib	0.1	0.10 ± 0.01	9.9	−0.5	0.10 ± 0.01	6.4	−0.7	97.3
	0.3	0.30 ± 0.03	9.7	1.5	0.30 ± 0.01	3.40	0.6	96.3
	100	94.14 ± 8.49	9.0	−5.9	99.23 ± 5.65	5.8	−0.8	96.0
	800	828.35 ± 88.76	10.7	3.5	824.18 ± 6.51	0.8	3.0	98.7

#### 3.2.5 Stability

Long- and short-term stability tests of the analytes were carried out by calculating the RE% and RSD% of QC samples under four different storage conditions. The stability test results for almonertinib are displayed in Table 4. Under different storage conditions, the RSD% is less than 13.1%, and the RE% is less than ± 11.3%. Based on the experimental results, almonertinib in plasma was stable under various circumstances (room temperature, 4°C refrigeration, freeze thawing and long-term cryopreservation).

**TABLE 4:** Summary of the stability of almonertinib in rat plasma under different storage conditions (*n* = 6).

Analyte	Concentration (ng/ml)	Room temperature		4°C		Three freeze-thaw		−80°C	
		RE (%)	RSD (%)	RE (%)	RSD (%)	RE (%)	RSD (%)	RE (%)	RSD (%)
Almonertinib	0.3	−7.2	12.9	11.3	9.4	4.6	7.1	−2.4	9.1
	100	4.1	13.1	−5.9	6.6	−5.5	5.9	−0.5	8.9
	800	−4.0	8.8	2.1	7.3	3.0	6.9	1.9	10.4

### 3.3 DDI Study

**FIGURE 3:**
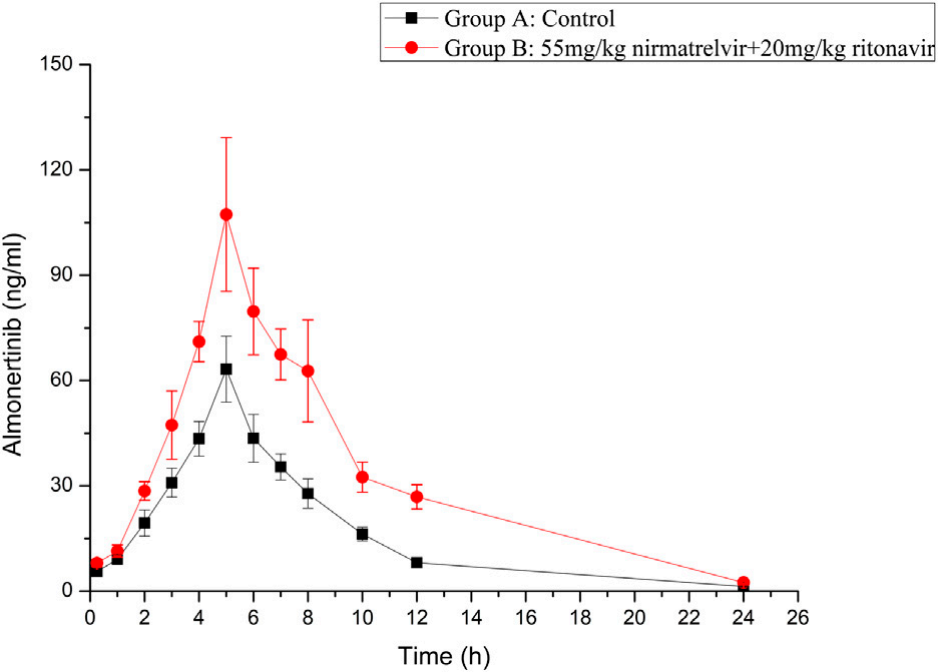
Mean plasma concentration-time curve of almonertinib in different treatment groups of rats. Group A: the control group (0.5% CMC-Na) and Group B: 55 mg/kg nirmatrelvir and 20 mg/kg ritonavir (n = 6, Mean ± SD).

The established UHPLC-MS/MS method was successfully used in the study of drug-drug interaction between almonertinib and Paxlovid in rats. The average plasma concentration curves of two groups at different time points after gavage administration of almonertinib (10 mg/kg) are presented in Figure 3. The main pharmacokinetic parameters of two groups are presented in Table 5.

**TABLE 5:** The main pharmacokinetic parameters of almonertinib in different treatment groups of rats. Group A: the control group (0.5% CMC-Na) and Group B: 55 mg/kg nirmatrelvir and 20 mg/kg ritonavir. (*n* = 6, Mean ± SD).

Parameters	Unit	Group A	Group B
AUC_(0-t)_	µg/L*h	385.33 ± 20.26	777.14 ± 47.80*
AUC_(0-∞)_	µg/L*h	389.85 ± 20.78	788.25 ± 50.22*
MRT_(0-t)_	h	6.92 ± 0.09	7.72 ± 0.22
MRT_(0-∞)_	h	7.30 ± 0.16	8.05 ± 0.20
t_1/2_	h	3.15 ± 0.82	3.40 ± 0.26
T_max_	h	5.00 ± 0.00	5.17 ± 0.41
CLz/F	L/h/kg	0.86 ± 0.65	2.23 ± 0.55*
C_max_	µg/L	63.23 ± 9.39	111.39 ± 16.12*

There are reports that co-administration of itraconazole 200 mg twice daily and single dose of 110 mg almonertinib significantly inhibited the metabolism of almetinib in healthy volunteers (Liu et al., 2022a). Itraconazole has been shown to be a potent CYP3A4 inhibitor (Brüggemann et al., 2009). The Paxlovid contains ritonavir, one kind of powerful CYP3A4 inhibitor (Sevrioukova and Poulos, 2014). Therefore, Paxlovid was chosen in combination with almonertinib to determine whether it would affect pharmacokinetics of almonertinib in rat. The results show that compared with the control group, the pharmacokinetic parameters of almonertinib, such as AUC_(0-t)_, AUC_(0-∞)_, CLz/F, and C_max_, were significantly increased (*p* < 0.05) when concomitantly used with Paxlovid. It shows that Paxlovid has obvious inhibiting effect on the metabolism of almonertinib, resulting in a significant increase in total systemic exposure to almonertinib. Therefore, extreme caution should be exercised when using almonertinib in combination with Paxlovid, the patients are more prone to severe adverse reactions due to elevated plasma levels of almonertinib. If the combination of the two drugs is inevitable, our results suggest that the dose of almonertinib should be reduced. Since the interaction study of almonertinib and Paxlovid was performed in a small number of rats, all results need to be validated in subsequent clinical trials.

## 4 Conclusion

This study established a fast, accurate, stable and facile ultra-performance liquid chromatography-tandem mass spectrometry method for the determination of almonertinib in rat plasma. The new method has been successfully applied to the drug interaction study of almonertinib and Paxlovid in rats. Paxlovid has a marked inhibitory effect on the metabolism of almonertinib, increasing the exposure of almonertinib. Considering the complexity of cancer patients, further human trials should be performed to verify the accuracy of animal studies.

## Data Availability

The original contributions presented in the study are included in the article/supplementary material, further inquiries can be directed to the corresponding authors.
